# A heuristic approach to handling missing data in biologics manufacturing databases

**DOI:** 10.1007/s00449-018-02059-5

**Published:** 2019-01-08

**Authors:** Jeanet Mante, Nishanthi Gangadharan, David J. Sewell, Richard Turner, Ray Field, Stephen G. Oliver, Nigel Slater, Duygu Dikicioglu

**Affiliations:** 1 Pembroke College, Cambridge, UK; 2 Department of Chemical Engineering and Biotechnology, University of Cambridge, Cambridge, UK; 3 Cell Sciences, Biopharmaceutical Development, MedImmune, Cambridge, UK; 4 Cambridge Systems Biology Centre, University of Cambridge, Cambridge, UK; 5 Department of Biochemistry, University of Cambridge, Cambridge, UK

**Keywords:** Biologics manufacturing data, Missing data, Imputation, Parameter recurrence, Data pre-processing

## Abstract

The biologics sector has amassed a wealth of data in the past three decades, in line with the bioprocess development and manufacturing guidelines, and analysis of these data with precision is expected to reveal behavioural patterns in cell populations that can be used for making predictions on how future culture processes might behave. The historical bioprocessing data likely comprise experiments conducted using different cell lines, to produce different products and may be years apart; the situation causing inter-batch variability and missing data points to human- and instrument-associated technical oversights. These unavoidable complications necessitate the introduction of a pre-processing step prior to data mining. This study investigated the efficiency of mean imputation and multivariate regression for filling in the missing information in historical bio-manufacturing datasets, and evaluated their performance by symbolic regression models and Bayesian non-parametric models in subsequent data processing. Mean substitution was shown to be a simple and efficient imputation method for relatively smooth, non-dynamical datasets, and regression imputation was effective whilst maintaining the existing standard deviation and shape of the distribution in dynamical datasets with less than 30% missing data. The nature of the missing information, whether Missing Completely At Random, Missing At Random or Missing Not At Random, emerged as the key feature for selecting the imputation method.

## Introduction

Biologics manufacturers have accumulated a large amount of data on biologics process development, scale-up and manufacturing operations to comply with the bioprocess development and manufacturing guidelines described by the Pharmaceutical Quality by Design initiative. Despite its existence, these large amounts of data on process parameters, culture properties and phenotypic characteristics of the cultivated organisms are not explored to its full extent. The field is expected to benefit significantly from the adaptation of approaches that combine model-based process optimisation with process-specific heuristics for enhancing yield and production efficiency. The extensive data recordings from the manufacturers could be used to assist Process Analytical Technology (PAT) implementation in biologics manufacture, provided that the database is exploited to its full potential [1].

One of the major challenges in mining biologics databases is associated with the fact that they contain highly heterogeneous data collected over long periods, from different projects, products and cell lines. The product type, the host cell type and consequently the bioprocess parameters exhibit changes over time. These biological variations, or how the biological variations manifest themselves affecting manufacturing conditions, lie in the immediate interest of biologics data mining, mainly due to the fact that recurrent patterns emerging from these data sets could be employed to improve existing processes, and even assist the implementation of smart bioprocessing systems, which have the ability to self-learn and self-adapt.

The heterogeneity of the database not only contributes to the wealth of information to be extracted, but also introduces challenges concerning its analysis. Compilation of data spread across years and different projects inherently introduces technical variations and inconsistencies; instrumentation systems change and/or receive updates, sampling is often not consistent across projects possibly due to limitations on instrumentation, and there is the inevitable variation due to the process operator, i.e., the human factor, and these obstacles result in missing/incomplete information to accumulate in databases. The way missing data are handled constitutes one of the major challenges in mining biologics manufacturing data since the method adopted to handle missing data has been shown to strongly influence the secondary analysis of the dataset [2, 3]. It is, therefore, imperative to choose a suitable method to deal with missing data to increase the accuracy of the predictions and interpretations made in the secondary analysis.

An understanding of the underlying pattern (also called the data structure, or the trends) was reported to be important to help us choose from an assortment of statistical methods available to address the different types of missing data [4]. There are different missing data mechanisms depending on whether the missing data depend on the observed values or the missing values themselves [5]. The structure of the biologics datasets indicates that they generally exhibit missing patterns across different days of the bioprocess, and that most of these missing values relate to parameters that are instrument-monitored and fetched automatically, except for a few, which are measured offline. Consequently, the biologics datasets appear to suffer from gaps that are missing at random. The possible analytical approaches to handle such data are (1) discarding incomplete cases; (2) imputing, i.e., filling in the missing data; or (3) analysing the incomplete data adopting a method that does not require a complete dataset [6]. Excluding large fractions of observations may introduce the risk of bias [7], and imputation was previously shown to improve classification accuracy in data analysis [8]. Gap filling by single or multiple imputations is a widely explored option in standard data pre-processing. Single imputation approaches employ a pre-determined method of imputation based on the nature of the dataset, and the gaps are filled in using the same method for the whole dataset every time, generating consistent results [9] and, therefore, deliver the simplest route.

This study focused on evaluating the efficacy of two single imputation methods—mean substitution and regression imputation to deal with missing information in two types of biologics manufacturing datasets: cell culture bioprocess harvest data and dynamic cultivation data. The imputed datasets were then exposed to secondary analysis via simple data processing to validate the performance of gap-filling strategies employed.

## Materials and methods

### Data

Two different types of bioprocess development and scale-up data from monoclonal antibody production using Chinese Hamster Ovary (CHO) cell lines provided by MedImmune were used in the study. The time series data, which consisted of daily parameter recordings from multiple culture batches that lasted for 14 days and the harvest day data were investigated separately. Time series data set consisted of readings of 14 different parameters from 75 cultures screened across 14 days of culture period, and harvest day data had recordings of 15 different parameters for the harvest days of 90 different cultures ranging between the 4th day to the 19th day post-inoculation. The parameters under investigation were viable cell density, elapsed culture time, culture volume, pH, total cell density, lactate, NH_3,_ glucose, average cell compactness, average cell diameter, glutamine, glutamate, Na^+^, K^+^, and osmolarity. In the interest of manufacturer proprietary rights, the parameters were anonymised as *A*–*O*, and the ‘Product’ row corresponds to final product titre for each batch (Table 1).

**Table 1:** Results of the non-normal robust *F* test

Parameters	Cubic model			Logarithmic model		
	Root mean square residual	*F* test against *y* = *d* model	*F* test against *y* = 0 model	Root mean square residual	*F* test against *y* = *d* model	*F* test against *y* = 0 model
*A*	13.1513	72359.3743	80166.6220	2.8468	1718339.4150	1718339.4150
*C*	69.3262	683.0890	831.2835	59.6001	1255.2270	1255.2270
*D*	76.2204	986.8539	1138.0420	67.4351	1556.4680	1556.4680
*E*	0.1385	0	892865.8000	0.1385	0	892865.8000
*F*	16.2188	57.6222	347.3065	15.5948	80.1238	405.0123
*G*	2.4787	4.5066	67.9761	2.4654	72.4435	72.4435
*H*	8.3052	0.7606	2593.0990	8.3060	0	2592.5060
*I*	0.0223	0	333463.8000	0.0223	0	333463.8000
*J*	1.2009	0.0657	37399.7500	1.0906	2440.5810	45390.0800
*K*	1.5372	15.3113	79.3399	1.4775	97.9238	97.9238
*L*	1.9222	0.0093	161.4175	1.9199	1.8973	162.2340
*M*	151.1273	1030.4960	1158.6530	49.8450	12235.0500	12235.0500
*N*	45.7868	671.2673	943.1314	20.6023	5453.9470	5453.9470
*O*	6.2970	548.3050	1754.2620	6.3071	1748.0530	1748.0530
Product	993.4056	860.9427	868.8166	996.9977	860.7497	860.7497

*F* stat critical value is 7 for all rows in both the models (*p* = 0.0001)

### Gap filling and statistical analysis

#### Gap filling in multivariate data

The harvest day dataset had 20.2% gaps that required imputation, and 36 of these 90 cultures had no missing values. The gaps in this complete dataset were imputed by mean substitution, and principal component analysis was carried out on the imputed and complete datasets to identify outliers, and any bias introduced by imputation.

A controlled mini-study was devised to investigate the effect of imputation by mean substitution on the dataset. Data points were randomly deleted from the subset of 36 cultures with no missing values to introduce 20.2% gaps equivalent to 20.2% of the dataset, and then the gaps were imputed with mean substitution. For validating the bias of the estimates, an analogous experiment was designed, in which the same gaps were filled by random number generation, using values that lie between the maximum and the minimum values that each variable took across the 36 datasets. The same was performed on simulated datasets with different percentages of gaps (1.4%, 19.1%, 25.7%, 28.4%, 33.1% or 66.4%).

The 3 datasets belonging to 36 cultivations with (1) no missing data, data with 20.2% gaps (2) imputed by random number generation, and (3) imputed by mean substitution were then employed in a test case for data analysis to evaluate the performance of data imputation strategy in subsequent data processing. The test case involved construction of mathematical models to describe the product titre in terms of the available bioprocess parameters. Symbolic regression (SR), which follows an evolutionary algorithm to construct the best fitting model for the data, was employed to construct these models [10]. 38 random combinations of parameter settings (population size—5, 50, 500, 5000; number of generations—5, 50, 500, 5000; maximum number of genes—1, 2, 3, 4, 10; maximum model depth—1, 2, 3, 4, 5, 10) for constructing the regression model were tested to evaluate the prediction success of the model. Randomly selected 67% of the dataset (24 cultivations) was employed to construct the models (training data), and the remaining 33% (12 cultivations) was spared to evaluate the predictive power of the constructed models (test data). The complexity of the constructed model was employed as the measure of its predictive success. A very complex model was highly successful in describing the training data whereas its predictive performance was poor on the test data, i.e., model overfitting. The difference between the prediction error of the model on the test data and the training data [testing error (TEE) − training error (TRE)] indicated model overfitting for highly positive values. Conversely, an over-simplistic model failed to adequately describe the training data, and was not employed to study the test data. To evaluate the effect of the fraction of data employed as training data on model performance, a similar analysis was conducted utilising 85% and 90% of the dataset as training data.

#### Gap filling in time series data

Regression imputation was used to fill in the 26.2% missing values in time series data to avoid significantly altering the standard deviation or the shape of the distribution [4]. Based on the nature of the distribution, polynomial regression (1) and logarithmic regression (2) were evaluated as potential candidates, with *y* being the nominal value for the parameter to be imputed, *x* being the day of sampling and *a, b, c*, and *d* being arbitrary constants:

1 $$Y=a{x^3}+b{x^2}+cx+d,$$

2 $$Y=a\left[ {\ln \left( {bx+c} \right)} \right]+d.$$

The root mean square of the residuals, which gives the difference between the observed parameter values and the calculated parameter values for the models, was then tested for a range of values, by assigning different values to the constants starting with an arbitrary non-zero value (in this case, 10). Having obtained the best fitting logarithmic and polynomial equations for all the parameters, non-normal robust *F* test was used to evaluate the significance of the best fit criterion employed to select the optimal model structure. A non-normal *F* test was selected for this evaluation owing to the non-normal distribution of the data and nonhomogeneous within-treatment variances. The *F* value was calculated by evaluating the correlation between MST (mean square between treatments—which is the square of the difference in means of two methods under consideration) and MSE (mean square error—which is the unbiased estimate of variance), and is given by the formula (3) [11]:

3 $$F={\text{MST}}/{\text{MSE.}}$$

The robust *F* test scores of all the parameters for both cubic and log models were compared against constant-only fit given by *Y* = *d*, where $d\in \mathbb{R}$. The better fitting model was then used for imputing the missing data points. The root mean square residual, which is the difference between observed value and the calculated value, was used in the comparison of the *F* test value against *Y* = *d*, and a measure of significance was given by *p* value < 0.0001 for the cubic and the logarithmic models.

A model-based clustering algorithm dedicated to the analysis of time series data was used as a subsequent data processing approach. The imputed dataset was employed to investigate how the temporal profiles of the operation parameters evolved through the progression of cultivation. The default merge and extension threshold settings of the tool, *m* = *e* = 0.5, were used [12].

## Results

### Gap filling in multivariate data

**Fig. 1:**
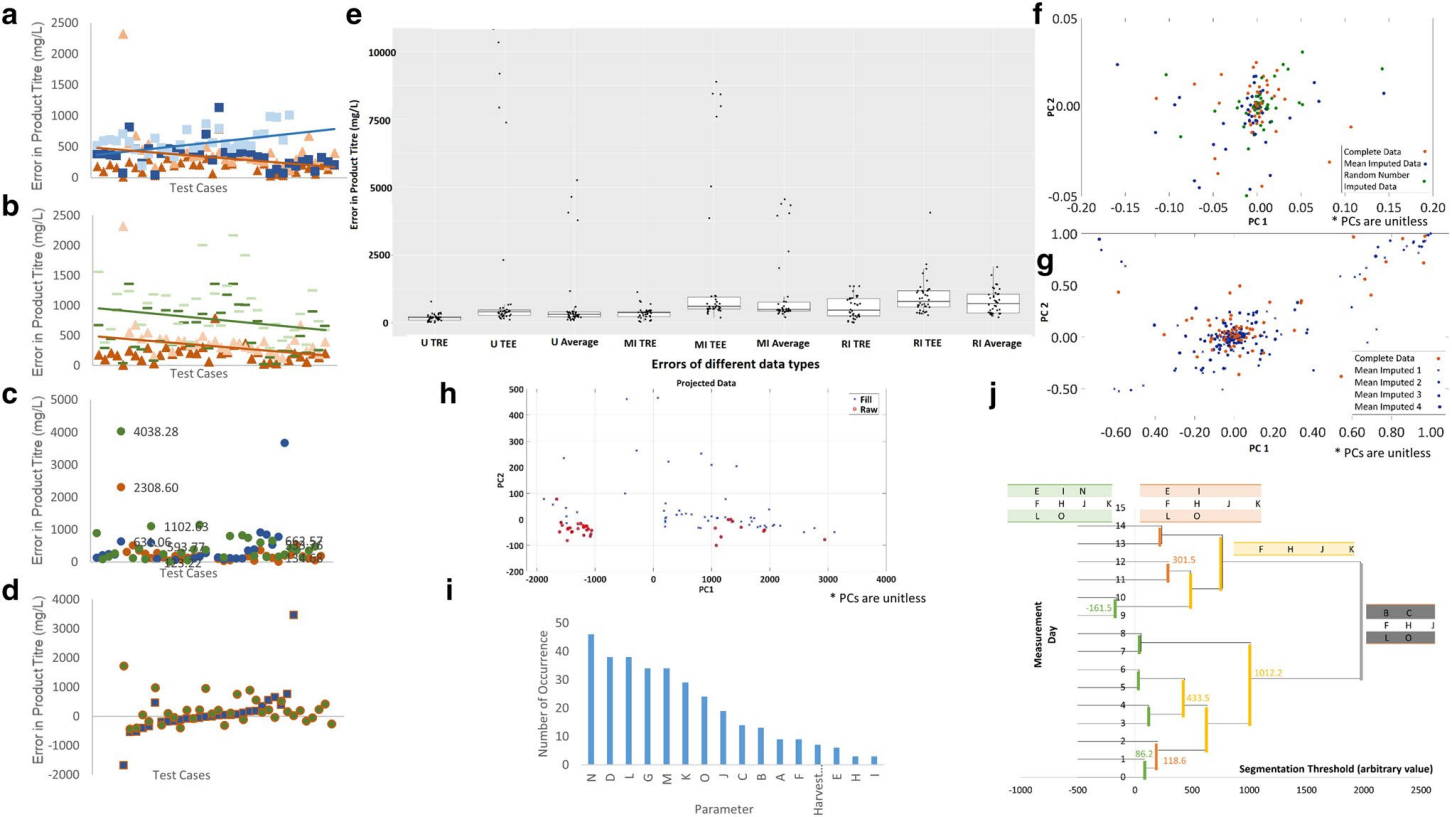
Performance evaluation to validate the efficacy of different methods of imputation. Predictive performance of SR analysis showing magnitude of errors (*Y*-axis) for 38 different combinations of SR parameters (*X*-axis) is provided in **a** and **b**. Straight lines denote the mean values. The ‘TEE − TRE’ test metric employed to evaluate model overfitting is provided in **c** in increasing values from left to right. Blue, green and orange refer to the mean substituted, randomly filled and unfilled datasets in **a–c**. TEE and the TRE are shown in lighter and darker shades of the same colour. The difference in the magnitude of overfitting with respect to the unfilled dataset is presented in **d**. Outlier values have been omitted for visualisation purposes in **a–d**. The magnitudes of the training and test errors as well as the average error distribution are displayed in **e** for the 38 test cases of unfilled and imputed data sets. Each box represents the interquartile range with upper line representing the third quartile, lower line the first quartile and centre line representing median of the distribution. *U* unfilled, *MI* mean imputed, *RI* random imputed datasets. Principal component analyses (PCA) for the complete data (in green), the mean imputed data (in red), and for the random imputed data (in blue) are projected on to the same plane (**f**). The results of the PCA for the complete data (in green) projected against four mirror cross-validation datasets of mean imputation (MI) (MI1, MI2, MI3, and MI4 in blue, magenta, red, and in black, respectively) (**g**). The results of PCA for the comprehensive mean-substituted dataset of 90 cultivations are shown in **h**. Red crosses denote the filled cultivations and the blue circles represent the raw data. PC1 and PC1 are represented in the abscissa and the ordinate, respectively. In all PCA analyses, the first two PCs explain more than 99% of the variance in their respective dataset (**f–h**). Parameter recurrence in the mean-substituted harvest day data for 90 cultivations is given in **i**. *X*-axis denotes the cultivation parameters and *Y*-axis denotes the number of times each parameter featured in the equations. Temporal segmentation clustering of the imputed time series data is shown in **j**. *X*-axis shows the segmentation threshold at which the consecutive time points form a single segment. Parameter clusters displayed in orange indicate clusters that formed early at the timescale, when many small time segments were formed. Moving from left to right on the *X*-axis, fewer time segments that span longer periods impose more stringent clustering conditions resulting in tighter clusters, and the parameters that exhibited similar patterns of behaviour over the whole period of culture decrease moving from green to orange, yellow and grey clusters. (Color figure online)

The performance of missing data imputation by mean substitution was evaluated against no imputation and against missing data imputation by random assignments in a mini-study investigating the effect of imputation in subsequent data processing. The main objective of implementing a successful imputation strategy is to yield a dataset which has similar performance to the complete dataset (i.e., no imputation) in data processing carried out post-imputation [13]. Regression models predicting culture titre from bioprocess parameters were constructed for data analysis as described above, and three measures were used to evaluate the success of missing data imputation method based on the three datasets tested: (1) the magnitudes of the testing error (TEE) and the training error (TRE), which are root mean squares errors (Fig. 1a, b); (2) the magnitude of the difference between the two error values, which assesses model overfitting (Fig. 1c); and (3) the magnitude of the difference between the overfitting of the imputed dataset and of the complete dataset (Fig. 1d). The prediction success of the models constructed using the complete dataset was observed to be more similar to those constructed using mean imputed data than using random substitution (32% vs 64%) (Fig. 1a, b). The models constructed using the dataset with randomly filled in gaps demonstrated an overfitting of 81% indicated by error magnitudes, dismissing the strategy as a potential gap-filling method (Fig. 1c), despite the absolute value of the difference between the TEE − TRE of the imputed dataset and the complete dataset being lower for random filling than for mean substitution across different test cases by 18% (Fig. 1d). An additional cross-validation was carried out by calculating a measure akin to the Predicted Residual Sum of Squares (PRESS) for the test data, where this parameter was calculated across ten simulated datasets by removing different samples and substituting them with model values. The results exhibited better fitting for mean imputed data than random number substitution, in comparison to the complete data (adjusted *R*^2^ = 0.9540 vs 0.8852) (Fig. 1e).

The simulated datasets and the complete dataset were further analysed for the distribution of parameters before data removal and after imputation by principal component analysis (PCA) (Fig. 1f, g). The distribution did not suggest any clustering, indicating the absence of any major bias introduced by the gap-filling strategy. Following the evaluation of the performance of mean substitution as a gap-filling strategy, the imputation method was employed for pre-processing the full harvest day data comprised of 90 cultivations. A projection of the raw data and mean imputed harvest day data on the same plane by PCA showed no evident clustering among the datasets, indicating that mean substitution introduced no additional bias (Fig. 1h), and thus could successfully be employed to pre-process datasets with relatively low percentage of gaps (this case 20.2%).

One of the success parameters, the magnitude of the difference between the two error values, was employed to determine the most suitable model construction parameters. Model overfitting was observed to increase with increasing either one of the generation number, the maximum number of genes or maximum depth of the model. Model construction parameters: population size: 500, number of generations: 500, the maximum number of genes: 4, and the maximum model depth: 2 were selected to yield only nominal overfitting for the dataset for which mean substitution was employed for imputation. As would be expected, increasing the size of the training set from 67% to 85 or 90% substantially reduced overfitting. A 27% increase in the training set size was sufficient to reduce the error in mean-substituted dataset by only 6%, whereas the improvement in prediction error was by 72.70% for random imputation. This indicated that a higher fraction of the dataset needed to be allocated to train the model when the gap filling is not informative about the data structure, as in the case of randomly filled data points.

The data processing approach employed in this analysis relied on principles of genetic programming to handle high-dimensional modelling problems with unknown model structure. Starting from a random population of individual models, the population evolves through a course of generations until an acceptable fit is achieved [10]. The evolutionary nature of the approach renders each analysis unique, and essentially unrepeatable. For this reason, the algorithm was allowed to run 50 times employing the optimal parameter settings to investigate recurring patterns. The standard deviation of the TEE and TRE values for 50 runs was spread across a range of 2–34% around the mean, indicating that although the actual models constructed in each run were unique, the predictive capability of the models remained within an acceptable limit. The parameters anonymised as D, G, L, M, and N were observed to be employed more frequently than others indicating that despite its heuristic nature, SR was able to nominate relevant culture parameters for attaining a mathematical representation of the product titre (Fig. 1i).

### Gap filling in time series data

A model-based imputation strategy was employed to address the missing data points in the time series dataset with the time course distribution of the cultivation parameters and the product roughly following logarithmic or third-degree polynomial trends. Each parameter and the product titre were analysed separately. The statistical evaluation of the robustness of both models for each culture parameter indicated that the logarithmic model performed better for all parameters, except for H, O, and the product titre (Table 1).

Both H and O were constant-only models, and thus would not be influenced by the choice of model. Even though the cubic model performed slightly better for imputation of the missing data in the product titre, both cubic and logarithmic models were shown to represent the trends in the data significantly better than a constant-only fit. Hence, for convenience, logarithmic regression model was employed for imputation across all parameters in the dataset.

Following gap filling, a data processing strategy which cannot work in the presence of gaps in the dataset was employed as an exercise to demonstrate the applicability of the approach. For this purpose, temporal segmentation clustering of the parameters [12] was conducted (Fig. 1j). This analysis allowed us to identify parameters, which displayed similar clustering patterns regardless of how many clusters were identified; many with fewer members and finer similarity relations; or only a few with many members and coarser similarity relations. Parameters anonymised as F, H and J were observed to cluster together regardless of the tightness in cluster similarity, indicating that these parameters displayed the same trend throughout the course of cultivation. Monitoring and tracking only one of these three parameters as a representative would be sufficient for PAT analytical purposes, and this information could potentially be critical in applications where there are limitations on the sample volume to be withdrawn from the culture.

## Discussion

This study demonstrated the implementation of two simple methods—mean substitution and regression imputation for handling missing data in biologics manufacturing databases—and showed that adopting these methods for a database of monoclonal antibody production using CHO cell lines did not introduce any bias in secondary analyses.

Understanding the rate and pattern of the missing data, its distribution, existing missing data mechanisms, and the nature of the data itself [14], emerged as imperative in the selection of a suitable gap-filling strategy. The biologics data employed in the study, despite spanning across several years and being collected from different cell lines producing different products, were relatively uniform, and of high quality. The number of critical process attributes monitored remained relatively constant over time; product titres and cellular physiologies remained within comparable ranges across different projects, and missing data constituted less than 30% of the data set. This allowed successful implementation of simple approaches, whose performance would be adversely affected by high proportions of data missing [15]. For cases where a known or a specific relationship exists, it could be better to apply prior knowledge about the expected behaviour of the data to construct a model for imputation. However, one should approach this method extremely cautiously, as such assumptions may lead to misleading results in exceptional cases where and if the data structure deviated from the “generally accepted and thus presumed” behaviour. The performance of the adopted gap-filling strategies was evaluated based on the extent of data that were actually missing in the original dataset; 20.2% of the harvest data and 26.2% of the time series data were missing. Regardless, the methodologies were initially tested on data with 1.4%, 19.1%, 25.7%, 28.4%, 33.1% or 66.4% gaps prior to this analysis, and the results were comparable for these datasets except for that where 66.4% of the data was missing.

Mean substitution on harvest day data and regression imputation on dynamic bioprocess data were both shown to perform adequately without substantially altering the standard deviation profile or shape of the data distribution. Secondary analysis of the harvest data via the use of predictive models revealed key parameters that contribute to variations in culture titre. Parameters that varied concurrently throughout the bioprocesses were identified in the secondary analysis of the dynamic data. Such coordinated behaviour among process parameters highlighted redundant measurements made, and could assist the design of future bioprocess experiments. All secondary analyses (i.e., data processing) could successfully be conducted without any interference from the adopted imputation strategy, demonstrating the elemental nature of selecting a suitable data pre-processing strategy before implementing complex methods such as predictive/descriptive statistical modelling and model-based inference in the mining of biologics data.
